# Prevalence of work-related musculoskeletal disorders among Iranian orthotists and prosthetists: A study on work-related quality of life

**DOI:** 10.33137/cpoj.v7i2.44064

**Published:** 2025-02-02

**Authors:** S Azizi, V Moradi, B Hajiaghaei, T Babaee

**Affiliations:** 1 Department of Orthotics and Prosthetics, School of Rehabilitation Sciences, Iran University of Medical Sciences, Tehran, Iran.; 2 Iran-Helal Institute of Applied Science and Technology, Tehran, Iran.

**Keywords:** Musculoskeletal Disorder, Work, Quality of Life, Orthotist, WRQoL, Rehabilitation, Musculoskeletal, Prosthetist, Iranian

## Abstract

**BACKGROUND:**

Work-related musculoskeletal injuries are prevalent globally and can impact both work efficiency and the quality of services offered to patients.

**OBJECTIVES:**

This study investigated the prevalence of work-related musculoskeletal disorders among Iranian orthotists and prosthetists and its relationship with work-related quality of life.

**METHODOLOGY:**

In this cross-sectional study, the Persian versions of the Nordic Musculoskeletal Questionnaire (NMQ) and Work-Related Quality of Life (WRQoL) scale were used to identify disorders of different body parts and occupational quality of life. A group of 263 orthotists and prosthetists were invited to complete the questionnaires using a simple random sampling method. The data from 188 respondents were analyzed. The association between WRQoL and the following variables: gender, work experience, age, presence of musculoskeletal disorder, and income level, was assessed using Spearman's rho correlation coefficient.

**FINDINGS:**

There was an 82% prevalence of work-related musculoskeletal disorders among the participants. The most common areas involved were the lower back (35%) and neck (28%). There was a statistically significant relationship between gender (*r_s_*(188) = 0.16, *p* = 0.031) and weight (*r_s_*(188) = 0.15, *p* = 0.045) and work-related musculoskeletal disorders, however, the strength of the correlation was very small. Moreover, the WRQoL had a statistically significant but small to negligible relationship with gender (*r_s_*(188) = 0.17, *p* = 0.016), work experience (*r_s_*(188) = 0.18, *p* = 0.011), age (*r_s_*(188) = 0.15, *p* = 0.039), and income (*r_s_*(178) = 0.17, *p* = 0.025). There was no significant relationship between work-related musculoskeletal disorders and the total score of WRQoL scale.

**CONCLUSIONS:**

While the prevalence of work-related musculoskeletal disorders is high among Iranian orthotists and prosthetists, the average WRQoL presents an opportunity for improvement. The findings indicate associations between work-related disorders and factors such as gender, work experience, age, and income, emphasizing the need for plans to improve working conditions and prevent these disorders. Conducting ergonomic assessments of workstations, increasing employee income, and implementing training programs that focus on proper lifting techniques, posture, and the use of ergonomic tools may help reduce work-related musculoskeletal disorders in orthotists and prosthetists.

## INTRODUCTION

Work-related musculoskeletal injuries are among the most severe occupational issues worldwide. Unfavorable body position during work is an essential factor in these injuries. According to the Health and Safety Executive, 38% of work-related problems are related to musculoskeletal disorders,^1^ the leading cause of more than half of work absences and about 50% of occupational diseases.^2^ These disorders are more frequently observed in occupations that require manual work, lifting heavy objects, or performing repetitive movements.^3^ Adjusting work habits, reducing working hours, or changing job types can help mitigate these issues.^4,5^ Demographic characteristics such as age, gender, and psychosocial factors are some predicting variables for musculoskeletal disorders.^6^ These disorders lead to waste of working days, increased costs, and human injuries and are the leading cause of disability.^7^ Work-related musculoskeletal disorders reduce work efficiency and production, affecting work-related quality of life (WRQoL).^8^ Issues related to WRQoL of employees affect their job satisfaction and their intention to stay on the job.^9^ Quality of Working Life (QoWL) focuses on different factors that affect a person's work experience and overall well-being. These include the physical work environment, the social atmosphere within the organization, conflicts related to work roles, job enrichment, fair compensation, flexible work hours, reward systems, the balance between work and family life, job security, and overall productivity and health.^10^ The connection between work-related stress and the broader idea of QoWL has been examined, revealing a link between job stress and burnout as significant negative factors affecting overall quality of life.^11^

Orthotists and prosthetists, as well as medical personnel, are at a higher risk of suffering from musculoskeletal disorders due to repetitive tasks, high workload, poor working conditions, psychological factors such as occupational stress, and work shifts.^12^ Manual handling of heavy plaster casts (sometimes more than 25 kg), limited resting time between activities, working in a limited workspace, and not having a helping hand to lift heavy objects imposes a high physical load on the musculoskeletal structure of these people.^13,14^ Therefore, the possibility of musculoskeletal disorders is high among orthotists and prosthetists due to long hours and heavy work, use of inappropriate and non-standard tools and machines, and lack of knowledge of the correct physical position.^15^ It has been reported that the prevalence of back disorders in manual labor is eight times higher than in occupations that do not involve manual work.^16^ In their study of 173 orthotists and prosthetists, Anderson et al. found that 76% of participants experienced musculoskeletal pain within the previous six months.^17^ Concerns related to workload, tight deadlines, and a poorly designed physical environment can increase fatigue and stress levels.^9^ Prosthetists and orthotists reported that the demands imposed by other healthcare staff and patients not only added to their workloads but also created unrealistic time constraints for task completion.^13^

Occupational pressures and injuries can negatively affect the services provided by healthcare personnel for society. This indicates the importance of and needs to consider the physical and mental health of the personnel working in health and treatment centers.^18^ Since the workstations in orthotics and prosthetics wards are non-adjustable and individuals of various heights and body dimensions must work at the same workstation, this situation can lead to the development of improper postures in the neck, shoulders, and back.^14^ They seem to be the main contributors to morbidity and disability in any workforce, affecting individuals' quality of life and work capacity.^19^ To the best of our knowledge, no studies have assessed WRQoL among orthotists and prosthetists.

Various studies have been conducted to investigate the prevalence of work-related musculoskeletal disorders in Iran. However, there are no accurate statistics on the prevalence of work-related musculoskeletal disorders and the quality of life of people working in this field of rehabilitation in Iran. This study aimed to determine the prevalence of work-related musculoskeletal disorders among orthotists and prosthetists and investigate its relationship with WRQoL.

## METHODOLOGY

This cross-sectional study was conducted from July 2023 to November 2023. Data was collected in person and online. To increase the generalizability of the study findings, the orthotists and prosthetists from all over Iran were invited to participate. Consequently, individuals working in Tehran, Iran, completed the questionnaires in person, while those situated other cities/provinces filled out the questionnaires online. It is important to note that the items in the questionnaires completed in person and online were exactly the same. Before completing the questionnaire, participants were given a consent form to sign. For the online version, an invitation message was sent via WhatsApp to individuals who had smartphones and internet access. The invitation included a brief description of the study and a link to the questionnaire. Consent was obtained by including a statement at the beginning of the online survey. The software used (https://porsline.ir) automatically removed respondents' phone numbers, ensuring that participants remained anonymous during the statistical analyses. The study protocol was approved by the research ethics committee of Iran University of Medical Sciences (Ref: # IR.IUMS.REC.1402.141, date: 24/05/2023).

The participants were asked to answer the Persian versions of the Nordic Musculoskeletal Questionnaire (NMQ)^20^ and the WRQoL scale.^21^ Completing these questionnaires took about seven minutes on average.

The NMQ is among the most commonly utilized questionnaires for evaluating work-related musculoskeletal disorders. It has proven to be highly reliable in assessing ergonomic hazards across different healthcare professionals.^22^ The WRQoL scale is widely recognized for its comprehensive approach to assessing various dimensions of factors across both work and non-work life domains.^23^ The NMQ and WRQoL scales are tools that broadly assess work-related musculoskeletal disorders and the quality of life of individuals across various professions.

The inclusion criteria included working as an orthotist or prosthetist at the time of the study,^24^ having at least one year of clinical work experience,^25^ having a smartphone, and having no previous musculoskeletal injuries during the past 12 months.^14^ To assess the participants' previous musculoskeletal injuries, the following question was asked: “Have you experienced injuries in your neck, shoulders, elbows, wrists, upper back, waist, thighs, knees, or ankles during the past 12 months?”. Those cases who did not complete the questionnaires were excluded.

### Nordic Musculoskeletal Questionnaire

This questionnaire is used in work-related healthcare studies to evaluate the prevalence of musculoskeletal disorders. The first part includes nine questions about pain and discomfort experienced in the past 12 months, the second part consists of nine questions about pain and discomfort experienced in the last seven days, and the third part consists of nine questions about reducing working hours and leaving the workplace due to pain and discomfort experienced in the past 12 months. All items are answered with “yes” or “no”. If participants answered “yes,” they were then asked whether the musculoskeletal pain had been present in the past 7 days (yes or no) and whether the musculoskeletal pain had hindered their ability to engage in regular work and daily activities over the past 12 months (yes or no). It is used to collect information on pain or discomfort in nine body parts: neck, shoulder, elbow, wrist, upper back, waist, thigh, knee, and ankle.^18,26^ The validity and reliability of its Persian version have been evaluated by Namnik et al. showing an acceptable internal consistency of more than 0.7, a standard error of measurement ranging 0.56 to 1.76, and a Kappa coefficient ranging 0.78 to 1).^20^

### The WRQoL scale

This questionnaire, designed by Van Laar and Easton in 2007, measures the QoWL.^27^ It consists of 24 items distributed into six subgroups to evaluate WRQoL. The scoring is based on a 5-point Likert scale. Twenty-three items evaluate six subgroups: general well-being, home-work interface, job-career satisfaction, control at work (referring to the degree of autonomy and influence an employee has over their work environment and tasks), working conditions, and stress at work. Item 24 evaluates satisfaction with the work-related quality of work. The full scale score of this questionnaire is obtained by calculating the average score from these six areas, ranging from 1 to 110, where a score of 1 to 71 indicates the lowest level of WRQoL and a score of 85 to 110 shows the highest level of WRQoL.^27^ Percentile equivalents of each WRQoL subscale are also categorized as low QoWL (score range of 10–30), medium QoWL (score ranges of 40–60), and high QoWL (score range of 70–99). Higher percentiles indicate a better QoWL. The validity and reliability of its Persian version have been investigated by Shabaninejad et al.^21^

#### Sampling method and sample size

A simple random sampling method was employed to include potential participants. The following formula^28^ was considered for sample size calculation:



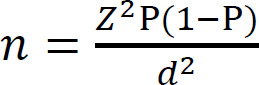



where n is the sample size, Z is the statistic corresponding to the level of confidence (99%), P is expected prevalence (60%),^29^ and d is precision (0.1); at least 159 participants were required. Considering that the average response rate for surveys among healthcare professionals is 53.3%,^30^ a total of 263 orthotists and prosthetists were invited to participate in this study.

#### Statistical Analysis

Descriptive data (means and standard deviations) and frequencies were reported for continuous and categorical variables, respectively. To check the normality of the data, the Kolmogorov-Smirnov test was run. The association between WRQoL and the following variables: gender, work experience, age, presence of musculoskeletal disorder, and income level, was assessed using Spearman's rho correlation coefficient. We considered the relationships as good to excellent, moderate to good, small, and small to no correlation if the r values were (r > 0.75), (0.50 < r < 0.75), (0.25 < r < 0.50), and (r < 0.25), respectively.^31^ The Mann-Whitney U test was used for data that did not follow a normal distribution, while the independent-sample t-test was applied to normally distributed data. These tests compared the mean values of WRQoL parameters between men and women. The Chi-square test was used to assess the relationships between categorical variables. All data were analyzed using the statistical package for social sciences (SPSS) software version 20. A threshold of 0.05 was considered as significant.

## RESULTS

A total of 263 orthotists and prosthetists were invited to participate in the study, either in-person or online. Of these, 207 completed the questionnaires, yielding a response rate of 78%. After applying the inclusion and exclusion criteria, the data from 188 participants (75 men and 113 women) were chosen and analyzed. Orthotists and prosthetists from various centers across different cities in Iran participated in this study. The mean of age, height, and weight of the participants were 31.4 ± 8.3 years old (range of 21 to 60 years old), 173.3 ± 9.5 cm (range of 150 to 197 cm), and 75.1 ± 14.6 kg (range of 45 to 110 kg). One hundred and four people were single, and 84 people were married. Their average daily working hours was 7.2 ± 2.8 hours (range of 1 to 15 hours), the average work experience was 7.4 ± 7.8 years (range of 1 to 34 years), and the average number of visitors per week was 26.2 ± 27.3 (range of 2 to 200 patients). As shown in **Table 1**, most participants worked in private clinics. Additionally, 40.4% of all participants engaged in activities outside of working hours, while 52.7% participated in sports activities.

**Table 1:** Percentage of participants by out-of-work activities and workplace type.

Workplace				Activities out of working hours		Sport activities	
Personal practice	Both government work environment and private clinic	Government work environment	Private clinic	No	Yes	No	Yes
26.8%	9.6%	8.5%	68.1%	59.6%	40.4%	44.7%	52.7%

A total of 141 orthotists and 47 prosthetists participated in the study. The prevalence rates of musculoskeletal disorders among the studied orthotists and prosthetists were 82% and 81%, respectively. 123 people (65.4%) stated that they felt pain, discomfort, burning, or numbness in different body parts during the last seven days.

Also, 77 people (41%) were forced to rest, reduce work activity, leave the workplace, or could not perform their activities at work or home during the past 12 months due to pain or discomfort (**Table 2**).

**Table 2:** Prevalence of musculoskeletal disorders in the past 12 months and 7 days, and the percentage of individuals requiring reduced activity or workplace leave.

Body segments	Reduced activity or leave the workplace frequency, n (%), in last 12 months	Musculoskeletal disorders frequency, n (%), in last 7 days	Musculoskeletal disorders frequency, n (%), in last 12 months
Neck	14 (7.4%)	40 (21.3%)	53 (28.2%)
Right shoulder	8 (4.3%)	17 (9%)	23 (12.2%)
Left shoulder	5 (2.7%)	11 (5.9%)	12 (6.4%)
Both shoulders	3 (1.6%)	11 (5.9%)	17 (9%)
Right elbow	2 (1.1%)	11 (5.9%)	13 (6.9%)
Left elbow	0 (0%)	0 (0%)	1 (0.5%)
Both elbows	1 (0.5%)	2 (1.1%)	3 (1.6%)
Right wrist/hand	9 (4.8%)	16 (8.5%)	30 (16%)
Left wrist/hand	1 (0.5%)	4 (2.1%)	6 (3.2%)
Both wrists/hands	2 (1.1%)	12 (6.4%)	21 (11.2%)
Upper back	7 (3.7%)	21 (11.2%)	33 (17.6%)
Lower back	14 (7.4%)	45 (23.9%)	66 (35.1%)
Right hip and thigh	6 (3.2%)	4 (2.1%)	8 (4.3%)
Left hip and thigh	8 (4.3%)	3 (1.6%)	8 (4.3%)
Both hips and thighs	11 (5.8%)	5 (2.7%)	7 (3.7%)
Right knee	3 (1.6%)	2 (1.1%)	8 (4.3%)
Left knee	2 (1.1%)	4 (2.1%)	10 (5.3%)
Both knees	6 (3.2%)	10 (5.3%)	23 (12.3%)
Right ankle and foot	0 (0%)	2 (1.1%)	6 (3.2%)
Left ankle and foot	2 (1.1%)	5 (2.7%)	10 (5.3%)
Both ankles and feet	9 (4.8%)	30 (16%)	40 (21.3%)

Results of Chi-square test revealed that there was a significant relationship between the participants' gender and musculoskeletal disorders in the last 12 months (Phi = 0.157, *p* = 0.031). There was a small to no relationship between participants' weight and musculoskeletal disorders in the last 12 months (*r_s_*(188) = 0.14, *p* = 0.045). The prevalence of these disorders was higher in men than women (89% versus 76%) and people with more weight. Decreased work activity, leaving the workplace, or being unable to perform activities at home or the workplace during the last 12 months had a statistically significant yet small to negligible relationship with the number of daily working hours (*r_s_*(188) = 0.22, *p* = 0.002).

Among the different areas of WRQoL, control at work (14.22 ± 1.88) had the highest mean, home-work interface (9.62 ± 2.53), and working conditions (9.84 ± 2.45) had the lowest mean, and the mean of WRQoL was 79.44 ± 10.70 (**Table 3**).

**Table 3:** Subscales scores of work-related quality of life scale in studied population.

Work related quality of life subscales	Stress at work	Working conditions	Control at work	Job-career satisfaction	Home-work interface	General well-being	Full-scale WRQoL
Mean ± SD	5.92 ± 1.94	9.84 ± 2.45	14.22 ± 1.88	20.41 ± 4.06	9.62 ± 2.53	19.41 ± 3.47	79.44 ± 10.70
Percentile equivalents*	Medium	Low	High	Medium	Low	Low	Medium

* The percentile ranges for low, medium, and high QoWL is 10–30, 40–60, and 70–99, respectively.

The WRQoL had a small to negligible but significant relationship with gender (*r_s_*(188) = 0.17, *p* = 0.016), work experience (*r_s_*(188) = 0.18, *p* = 0.011), age (*r_s_*(188) = 0.15, *p* = 0.039), and income (*r_s_*(178)= 0.17, *p* =0.025) (**Table 4**).

**Table 4:** Relationship between work-related quality of life subscales and parameters of interest.

WRQoL subscales	The average number of visits per week		Income		Age		Work experience	
	*p*	*r*	*p*	*r*	*p*	*r*	*p*	*r*
General well-being	**0.04**	0.13	0.77	0.21	**0.01**	0.26	**0.01**	0.24
Home-work interface	0.41	−0.06	**0.02**	0.16	0.29	0.07	0.39	0.06
Job-career satisfaction	0.21	−0.09	0.20	0.09	0.12	0.11	**0.04**	0.15
Control at work	0.66	0.03	0.22	0.09	0.85	−0.01	0.95	−0.04
Working conditions	0.83	0.01	0.33	0.07	0.21	0.09	0.03	0.15
Stress at work	0.4	0.06	0.28	0.08	**0.03**	−0.15	**0.03**	−0.15
Full scale WRQoL	0.63	0.03	**0.02**	0.16	**0.03**	0.15	**0.01**	0.18

The WRQoL was higher in women than in men. All areas of the quality of work life had a higher mean in women than men, except stress at work (**Table 5**).

**Table 5:** Comparing the results of work-related quality of life subscales between men and women.

WRQoL subscales	Men		Women		*p*
	Quality of work life	Mean ± SD	Quality of work life	Mean ± SD	
General well-being	Low	18.78 ± 3.64	Medium	19.83 ± 3.31	**0.04**
Home-work interface	Low	9.54 ± 2.80	Low	9.67 ± 2.35	0.51
Job-career satisfaction	Medium	19.52 ± 4.50	Medium	21.00 ± 3.64	**0.01**
Control at work	High	13.77 ± 2.20	High	14.53 ± 1.57	**0.01**
Working conditions	Low	9.56 ± 2.83	Low	10.02 ± 2.15	0.26
Stress at work	Medium	5.98 ± 1.87	Medium	5.88 ± 1.99	0.47
Full scale WRQoL	Medium	77.17 ± 12.45	Medium	80.95 ± 9.12	0.01

There was no statistically significant relationship between general musculoskeletal disorders and overall WRQoL. Still, these disorders were significantly related to general well-being (*r_s_*(188) = 0.16, *p*= 0.023).

## DISCUSSION

This study investigated the prevalence of work-related musculoskeletal disorders among Iranian orthotists and prosthetists and its relationship with WRQoL. The results showed a high prevalence of work-related musculoskeletal disorders among Iranian orthotists and prosthetists who had a minimum one year of clinical experience. 82% of the studied orthotists and prosthetists had experienced pain in different body parts during the past 12 months. This was in line with Anderson et al.'s study on Australian orthotists and prosthetists (80%).^24^ Still, the prevalence of musculoskeletal pain found in this study was higher than that reported by Farahmand et al., who noted that 60% of orthotists experienced pain in their shoulders, elbows, and wrists over the last year. In contrast, the prevalence of musculoskeletal pain among prosthetists was 33%.^29^ Notably, this study found that the prevalence of musculoskeletal pain was the same for both orthotists and prosthetists.

Some factors contributing to musculoskeletal disorders in orthotists and prosthetists include the poor design of tools and machinery, long working hours using them, manual lifting and moving of heavy objects (such as molded plaster) without assistance, working with vibrating devices (like milling machines and drills) and performing repetitive tasks.^29^ Findings from this study showed that pain is most prevalent in the back (35.1%), neck (28.2%), and ankles and feet (21.3%), with lower back pain being the most common musculoskeletal disorder among orthotists and prosthetists. In the study by Anderson et al.,^16^ it was reported that the prevalence of disorders in the lumbar region is eight times higher in jobs involving manual activity compared to jobs that do not require manual activity. The manual handling of heavy plaster molds for various processes such as mold correction, lamination, thermoplastic mold production, and the preparation of negative and positive plaster molds, as well as filling the molds, is common in orthotic and prosthetic fabrication facilities. These factors may explain the high prevalence of back pain among orthotists and prosthetists.

There seems to be a small to negligible yet statistically significant relationship between gender and musculoskeletal disorders, as the findings of this study show that the prevalence of these disorders is higher in men. This may be due to the fact that men tend to be more inclined to perform heavier lifts. The results of this study are in line with Farahmand et al.'s findings.^29^ However, in other studies, such as those by Anderson et al. on Australian orthotists and prosthetists,^24^ Rahimi et al. on Iranian physiotherapists,^32^ Nazari et al. on occupation therapists,^33^ and Keyhani et al. on Iranian dentists,^34^ the prevalence of work-related musculoskeletal disorders was higher in women. Additionally, there was a small to negligible but significant relationship between weight and feeling pain and discomfort due to musculoskeletal disorders over the past 12 months. Still, this study did not find a significant relationship between these disorders and other demographic characteristics such as age, height, work experience, income, and working hours. It is important to note that, due to the small to negligible relationship between gender and musculoskeletal disorders, these results should be interpreted with caution.

Only 52.7% of the studied orthotists and prosthetists engaged in regular sports activities and showed little tendency to reduce their activity or take rest when they felt pain and discomfort. Additionally, about 48% of people who experienced pain and discomfort in the last 12 months reduced their activity during work. Decreased work activity, leaving the workplace, or an inability to perform tasks at home or the workplace during the last 12 months was more common in women (57%), although this difference was not significant. These results are similar to those found in Rahimi et al.'s study on Iranian physiotherapists.^32^

The total score of the WRQoL questionnaire among the studied orthotists and prosthetists was 79.44, which, based on the proposed percentile equivalents, falls within the average range. Among its different aspects, control at the workplace was more favorable, and working conditions and the relationship between home and work were less favorable. Also, the mean WRQoL score was significantly related to gender and was higher in women. The average quality of life for female orthotists and prosthetists was higher than that of their men counterparts in all aspects except stress at work. There was a significant difference in general well-being, control at work, and job-career satisfaction between men and women.

Regarding the demographic characteristics and WRQoL, there was a small to negligible but significant relationship between age, gender, work experience, income, and overall quality of life. The WRQoL was higher in women and participants of older ages, higher income, and greater work experience. The reasons for this could include increased job satisfaction, better control of the work environment, and reduced stress because of more work experience, age, and income. Arab et al. found no significant difference between age, sex, employment status, working hours, and WRQoL. However, specialist physicians with the least work experience had the highest WRQoL.^35^ In Abbasi et al.'s study on nurses, age and work experience had an inverse relationship with WRQoL.^36^ In light of the small to negligible relationship between the demographic characteristics and WRQoL in this study, it is important to interpret these results cautiously.

In this study, there was no significant relationship between the overall score of the WRQoL and work-related musculoskeletal disorders. However, a significant relationship existed between WRQoL and musculoskeletal disorders in different body areas. Regarding each subdomain of WRQoL, results of this study revealed a small to no relationship between general well-being and musculoskeletal disorder. It should be noted that the overall WRQoL score is calculated by summing the values of its six subdomains: general well-being, home-work interface, job-career satisfaction, control at work, working conditions, and stress at work. Consequently, the score of each subdomain influences the total score of the questionnaire. However, general well-being is an independent subdomain; its score is not affected by the scores of the other subdomains. This independence may explain the lack of a significant relationship between overall WRQoL and musculoskeletal disorders, as fluctuations in other areas do not impact the general well-being score.

### Limitations

This was a cross-sectional study and, therefore, cannot show the conditions of the participants over time. Also, the questions were asked about past events to evaluate the working conditions of participants (for example, in the past 12 months or seven days). Thus, they may not have answered the questions accurately due to the effects of memory over time. The WRQoL scale is designed without a distinction between employees and employers, so the responses might not be accurate for some questions about the work environment and the employer. Moreover, in this study, there was no age limit for inclusion. Most participants were under 50 years old, with 11 participants aged between 50 and 60 years. The statistical analysis revealed a small to no relationship between WRQoL and participants' age. Further research is needed to assess the rate of musculoskeletal injuries among orthotists and prosthetists across different age classifications and marital statuses, utilizing a sufficient sample size. In addition, this study included orthotists and prosthetists with at least one year of clinical work experience. Future investigations are needed to assess the prevalence of work-related musculoskeletal disorders among novice orthotists and prosthetists with less than one year of clinical experience.

## CONCLUSION

There appears to be a high prevalence of work-related musculoskeletal disorders among Iranian orthotists and prosthetists. Symptoms of pain in the back and neck are the most common issues. The Iranian orthotists and prosthetists seem to have an average WRQoL, which is higher in women and individuals with more work experience. Addressing the high incidence of musculoskeletal disorders and enhancing the WRQoL for orthotists and prosthetists may lead to improved health outcomes and job performance in this essential healthcare sector.

## DECLARATION OF CONFLICTING INTERESTS

The authors declare that there is no conflict of interest.

## AUTHOR CONTRIBUTION

- **Sarina Azizi:** Conception and design; Data acquisition; Analysis and interpretation; Drafting the article; Revision and final approval of the manuscript.
- **Vahideh Moradi:** Conception and design; Review and final approval of the manuscript.
- **Behnam Hajiaghaei:** Conception and design; Review and final approval of the manuscript.
- **Taher Babaee:** Conception and design; Analysis and interpretation, Revision and final approval of the manuscript.

## SOURCES OF SUPPORT

No external support was obtained for this project.

## ETHICAL APPROVAL

The study protocol was approved by the research ethics committee of Iran University of Medical Sciences (Ref: # IR.IUMS.REC.1402.141, date: 24/05/2023).

## References

[1] Saraji J, Hosseini M, Shahtaheri S, Golbabaei F, Ghasemkhani M. Evaluation of ergonomic postures of dental professions by rapid entire body assessment (REBA), in Birjand, Iran. J Dent Med. 2005;18(1):61–7

[2] Azaroff LS, Levenstein C, Wegman DH. Occupational injury and illness surveillance: conceptual filters explain underreporting. Am J Public Health. 2002;92(9):1421–9. DOI: 10.2105/ajph.92.9.1421 12197968 PMC1447253

[3] Vieira ER, Svoboda S, Belniak A, Brunt D, Rose-St Prix C, Roberts L, et al. Work-related musculoskeletal disorders among physical therapists: An online survey. Disabil Rehabil. 2016;38(6):552–7. DOI: 10.3109/09638288.2015.1049375 26007284

[4] Atia DT, Abdelazeim FH, Radwan H. Impact of work-related musculoskeletal disorders on Egyptian pediatric physical therapists: One-year follow-up study. Trends Applied Sci Res. 2015;10(3):175. DOI: 10.3923/tasr.2015.175.182

[5] Campo M, Weiser S, Koenig KL, Nordin M. Work-related musculoskeletal disorders in physical therapists: A prospective cohort study with 1-year follow-up. Phys Ther. 2008;88(5):608–19. DOI: 10.2522/ptj.20070127 18276935 PMC2390722

[6] Deeney C, O'Sullivan L. Work related psychosocial risks and musculoskeletal disorders: Potential risk factors, causation and evaluation methods. Work. 2009;34(2):239–248. DOI: 10.3233/WOR-2009-0921 20037236

[7] Da Costa BR, Vieira ER. Risk factors for work-related musculoskeletal disorders: A systematic review of recent longitudinal studies. Am J Ind Med. 2010;53(3):285–323. DOI: 10.1002/ajim.20750 19753591

[8] Vieira ER, Kumar S. Working postures: A literature review. J Occup Rehabil. 2004;14:143–159. DOI: 10.1023/b:joor.0000018330.46029.05 15074366

[9] Gurses AP, Carayon P, Wall M. Impact of performance obstacles on intensive care nurses' workload, perceived quality and safety of care, and quality of working life. Health Serv Res. 2009;44(2p1):422–443. DOI: 10.1111/j.1475-6773.2008.00934.x 19207589 PMC2677047

[10] Chen WS, Haniff J, Siau CS, Seet W, Loh SF, Abd MH, et al. Psychometric properties of the Malay work-related quality of life (WRQoL) scale in Malaysia. World J Soc Sci Res. 2014;1(1)

[11] Killian JG. Career and technical education teacher burnout: Impact of humor-coping style and job-related stress. Southern Illinois University at Carbondale; 2004

[12] Chung YC, Hung CT, Li SF, Lee HM, Wang SG, Chang SC, et al. Risk of musculoskeletal disorder among Taiwanese nurses cohort: A nationwide population-based study. BMC Musculoskelet Disord. 2013:14:144. DOI: 10.1186/1471-2474-14-144 23617330 PMC3637823

[13] Anderson S, Stuckey R, Oakman JR. Prosthetists’ and orthotists’ experience of their work and workspace–characterising the physical and organisational environment: Focus group findings. Prosthet Orthot Int. 2016;40(6):703–712. DOI: 10.1177/0309364615592702 26205672

[14] Salmani Nodooshan H, Koohi Booshehri S, Daneshmandi H, Choobineh A. Ergonomic workplace assessment in orthotic and prosthetic workshops. Work. 2016;55(2):463–470. DOI: 10.3233/WOR-162401 27689584

[15] Andersson GB. Epidemiological features of chronic low-back pain. Lancet. 1999;354(9178):581–585. DOI: 10.1016/S0140-6736(99)01312-4 10470716

[16] Anderson SP, Oakman J. Allied health professionals and work-related musculoskeletal disorders: A systematic review. Saf Health Work. 2016;7(4):259–267. DOI: 10.1016/j.shaw.2016.04.001 27924228 PMC5127976

[17] Anderson S, Weale V, Stuckey R, Oakman J. Work-related musculoskeletal pain in prosthetists and orthotists: A comparison between Australia and other countries. Prosthet Orthot Int. 2021;45(6):538–543. DOI: 10.1097/PXR.0000000000000051 34759257

[18] Valipour F, Mohammadian MS, Yahyaei E, Shokri S, Ahmadi O. Assessment of the staff working posture using REBA & ROSA methods in a military hospital. Health Res. 2016;1(3):167–72. DOI: 10.18869/acadpub.hrjbaq.1.3.171

[19] Centers for Disease Control and Prevention (CDC). About ergonomics and work-related musculoskeletal disorders [Internet]. Atlanta, GA: National Institute for Occupational Safety and Health (NIOSH); [cited 2024 Oct 8]. Available from: https://www.cdc.gov/niosh/ergonomics/about/index.html

[20] Namnik N, Negahban H, Salehi R, Shafizadeh R, Tabib MS. Validity and reliability of Persian version of the Specific Nordic questionnaire in Iranian industrial workers. Work. 2016;54(1):35–41. DOI: 10.3233/WOR-162268 26967033

[21] Shabaninejad H, Arab M, Rashidian A, Zeraati H, Bahrami S. Quality of working life of family physicians in Mazandaran. Hakim J. 2012;15(2):178–184

[22] Kakaraparthi VN, Vishwanathan K, Gadhavi B, Reddy RS, Tedla JS, Alshahrani MS, et al. Clinical application of rapid upper limb assessment and nordic musculoskeletal questionnaire in work-related musculoskeletal disorders: A bibliometric study. Int J Environ Res Public Health. 2023;20(3):1932. DOI: 10.3390/ijerph20031932 36767293 PMC9914731

[23] Garzaro G, Clari M, Donato F, Dimonte V, Mucci N, Easton S, et al. A contribution to the validation of the Italian version of the work-related quality of life scale. Med Lav. 2020;111(1):32–45. DOI: 10.23749/mdl.v111i1.8570 32096771 PMC7809961

[24] Anderson S, Stuckey R, Oakman J. Work-related musculoskeletal injuries in prosthetists and orthotists in Australia. Int J Occup Saf Ergon. 2021;27(3):708–713. DOI: 10.1080/10803548.2018.1485335 29893181

[25] Kuorinka I, Jonsson B, Kilbom A, Vinterberg H, Biering-Sørensen F, Andersson G, et al. Standardised Nordic questionnaires for the analysis of musculoskeletal symptoms. Appl Ergon. 1987;18(3):233–7. DOI: 10.1016/0003-6870(87)90010-x 15676628

[26] Dembe AE. The social consequences of occupational injuries and illnesses. Am J Ind Med. 2001;40(4):403–17. DOI: 10.1002/ajim.1113 11598991

[27] Van Laar D, Edwards JA, Easton S. The work-related quality of life scale for healthcare workers. J Adv Nurs. 2007;60(3):325–33. DOI: 10.1111/j.1365-2648.2007.04409.x 17908128

[28] Pourhoseingholi MA, Vahedi M, Rahimzadeh M. Sample size calculation in medical studies. Gastroenterol Hepatol Bed Bench. 2013;6(1):14–7 24834239 PMC4017493

[29] Farahmand B, Mohammadi M, Hassanbeygi B, Mohammadi M, Saeedi H, Bagherzadeh Cham M. Ergonomic evaluation of working conditions in orthotists and prosthetists by rapid entire body assessment (REBA). Function Disabil. 2020;3(1):169–78. DOI: 10.32598/fdj.3.22

[30] Meyer VM, Benjamens S, Moumni ME, Lange JFM, Pol RA. Global overview of response rates in patient and health care professional surveys in surgery: A systematic review. Ann Surg. Jan 1 2022;275(1):e75–e81. DOI: 10.1097/sla.0000000000004078 32649458 PMC8683255

[31] Portney LG. Foundations of Clinical Research: Applications to Evidence-Based Practice. 4th ed. Philadelphia: F.A. Davis; 2020. Available from: https://fadavispt.mhmedical.com/book.aspx?bookID=2885

[32] Rahimi F, Kazemi K, Zahednejad S, López-López D, Calvo-Lobo C. Prevalence of work-related musculoskeletal disorders in Iranian physical therapists: A cross-sectional study. J Manipulative Physiol Ther. 2018;41(6):503–507. DOI: 10.1016/j.jmpt.2018.02.003 30098820

[33] Nazari H, Hosseini Mahjoob H, Tapak L, Mortazavi SS. Prevalence of work-related musculoskeletal disorders and injuries in occupational and physical therapists and its comparison. Iran Rehabil J. 2017;15(1):31–6. DOI: 10.18869/nrip.irj.15.1.31

[34] Kahyani ZA, Karimi M, Amiri M, Mosharraf S, Broujeni HR. Determination of risk factors for musculoskeletal disorders and corrective priorities to perform the work in dental careers by posture analysis using REBA in Shahrekord. Epidemiol health system j. 2019;6(3):92–5. DOI: 10.15171/ijer.2019.17

[35] Arab M, Shabaninejad H, Rashidian A, Rahimi A, Purketabi K. A survey on working life quality of specialists working in affiliated hospitals of TUMS. Hospital J. 2013;11(4)

[36] Abbasi M, Zakerian A, Akbarzade A, Dinarvand N, Ghaljahi M, Poursadeghiyan M, et al. Investigation of the relationship between work ability and work-related quality of life in nurses. Iran J Public Health. 2017;46(10):1404 29308385 PMC5750353

